# How do determinants of health relate to children’s quality of life? A cross-sectional study in Izmir, Turkey

**DOI:** 10.1017/S1463423623000397

**Published:** 2023-09-14

**Authors:** Hilal Duzel, Isil Ergin, Raika Durusoy

**Affiliations:** 1 Department of Public Health, Dokuz Eylul University Faculty of Medicine, Izmir, Turkey; 2 Department of Public Health, Ege University Faculty of Medicine, Izmir, Turkey

**Keywords:** child health, quality of life, social determinants, socioeconomic factors

## Abstract

**Aim:**

This study aims to determine health-related quality of life (QoL) and the related factors from the perspective of social determinants of health among children.

**Background:**

Childhood is the most intense period of life, and environmental factors surrounding children, as well as individual lifestyle factors, are related to the child’s physical and mental well-being. To our knowledge, there is a lack of studies evaluating the relationship between determinants of health and the QoL of healthy children in general.

**Methods:**

This cross-sectional study was executed in the Bayrakli district of Izmir city. Stratified clustered sampling was used including 24 schools and 3367 7th-grade children, and 1284 students were targeted (50% prevalence, 95% CI, %5 margins of error, 2.25 design effect, and 20% replacement). The response rate was 84.9% (*n* = 1090). The Turkish KID-KINDL Health-Related Quality of Life Questionnaire for Children was used to assess QoL. Independent variables were examined in four layers using Dahlgren’s Determinants of Health Model: basic characteristics, lifestyle factors, family characteristics, and life conditions.

**Results:**

The mean QoL score was 71.3 ± 12.6. Our study explained 31.7% of the variance in QoL. Higher QoL scores were associated with better health status, perceived academic achievement, normal/thin body perception, physical activity (PA), and adequate sleep duration. Living with both parents and having fewer siblings positively influenced QoL. Moreover, the presence of structural problems in the household and poorer health perceptions were associated with lower QoL scores (*P* < 0.05) This study highlighted the multifaceted nature of QoL in Turkish children, revealing the importance of various determinants of health. The results show that in order to improve the general well-being of this population, interventions and policies are required that concentrate on elements including health status, academic accomplishment, body perception, physical activity, family structure, and living situations.

## Introduction

World Health Organization Quality of Life Group (WHOQOL) defines the quality of life (QoL) as ‘individuals’ perception of their position in life in the context of culture and value systems in which they live and about their goals, expectations, standards, and concerns’ (Saxena and Orley, 1997). Childhood is the most intense period of life regarding emotional, cognitive, and physical development. Children are socially inadequate and dependent on adults most of the time for their many needs in life. Therefore, they can interpret the various events they encounter differently from adults (Eiser and Seamark, 1999). It is crucial to evaluate adverse conditions’ effects on child health correctly (Eiser, 1997; Eiser *et al.,* 2000). Child-specific health-related QoL measures are valuable tools for this evaluation.

Determinants of health vary throughout the life course. According to Dahlgren, while planning political actions for improving health outcomes, interventions should be made regarding these structural levels: individual lifestyle factors, social and community networks, living and working conditions, and general socioeconomic, cultural, and environmental conditions (Dahlgren and Whitehead, 2007). Environmental factors surrounding children, as well as individual lifestyle factors, are related to the child’s physical and mental well-being (Cohen *et al.,* 2010).

Studies have evaluated QoL in children in many different ways in recent years. However, many of them reported how various diseases affected children. There is a lack of studies evaluating the relationship between determinants of health and the QoL of healthy children in general. This study aims to determine health-related QoL and the related factors from the perspective of social determinants of health among children attending seventh grade at secondary schools in the Bayrakli district of Izmir City, Turkey.

## Materials and methods

### Setting and participants

This cross-sectional study was executed in the Bayraklı district of Izmir between January and December 2016. Izmir, the third most populated city in Turkey, is located in the very west and relatively the most modernized and developed part of the country. Bayraklı, with a population of 312,263, is the sixth most crowded district in Izmir and consists of 24 neighborhoods with different socioeconomic levels. The study was conducted among 7th-grade secondary school children. Stratified clustered sampling was used, and 47 classes from 24 schools (21 public, and3 private) were included. Among the 3367 7th-grade children, 1284 students were aimed to be reached (50% prevalence (Gürsel *et al.,* 2014), 95% confidence interval, %5 margins of error, 2.25 design effect, and 20% replacement). The minimum number of students needed to be included in the study was calculated in the stratification, considering the number of students in each school. Enough classes were included in the study to meet this threshold. Therefore, it can be said that all schools in the district are well represented in the study. Inclusion criteria were being at school on the day of the research and the absence of physical or mental barriers to participation, and 1090 students took part (response rate was 84.9%).

### Variables

The dependent variable of the study was the health-related QoL. The Turkish KID-KINDL Health-Related Quality of Life Questionnaire for Children was used for evaluation (Cronbach’s alpha > 0.75) (Eser *et al.,* 2008). KID-KINDL had six dimensions: physical, emotional well-being, self-esteem, family, friends, and school, and each dimension had four questions rated between one and five points. The higher scores indicated better QoL status.

**Figure 1.**
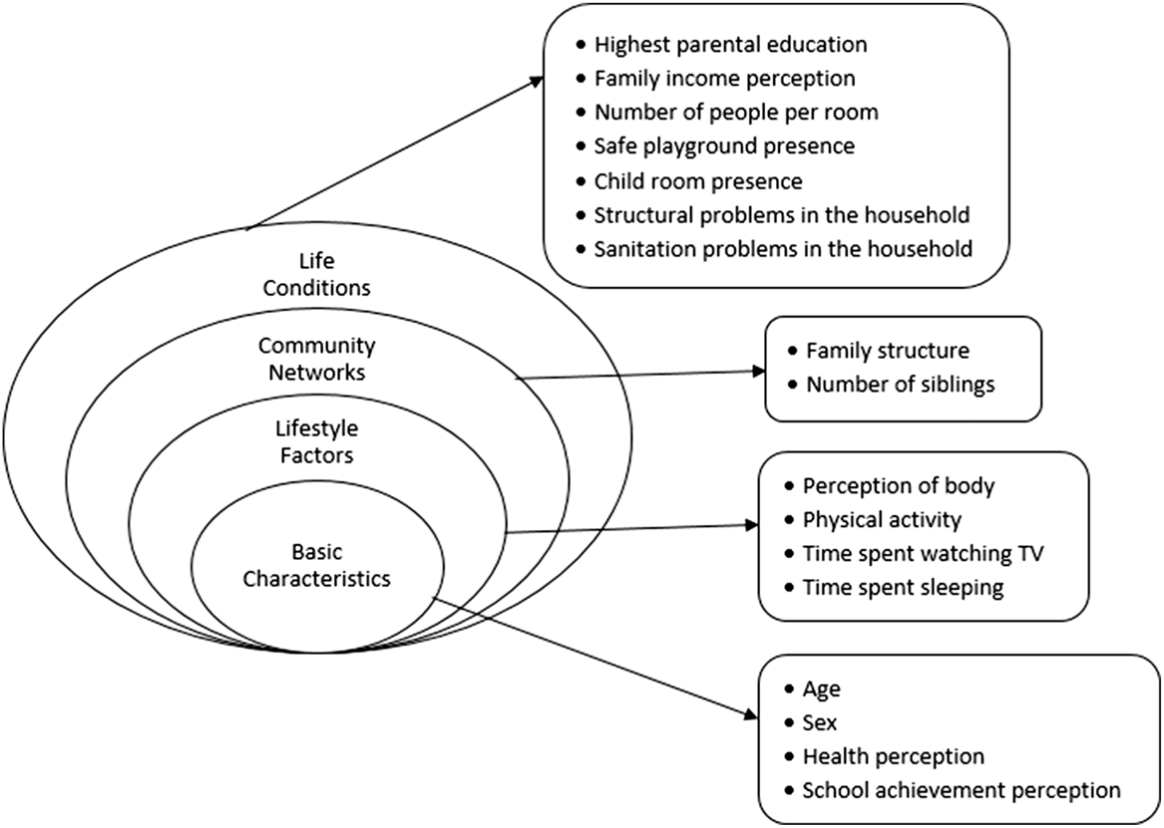
Four layers of independent variables designed in accordance with Dahlgren’s Determinants of Health Model (‘The Rainbow Model’).

The independent variables were examined in four layers derived using Dahlgren’s Determinants of Health Model (basic characteristics of the child, lifestyle factors, community networks, and living conditions) and are shown in Figure 1.

The first layer (basic characteristics) included age, sex, health status, and perceived academic achievement. Health status was determined according to the children’s perception of their health status and grouped as very well, good, and bad regarding their response to ‘How do you define your health generally?’. The perceived academic achievement was used as an indirect measure of cognitive level. Responses to the ‘How well do you think you are at school?’ question was grouped as near the top, about the middle, and near the bottom (Clark *et al.,* 2012).

The second layer (lifestyle factors) included body perception, physical activity (PA), time spent across TV, and time spent sleeping. Body perception was grouped as overweight, normal, or underweight using the answers to the ‘Considering your own body, which one of the following fits you most?’ question. Moderate- to vigorous-intensity PA definitions were stated and asked, ‘How many days did your PA last at least 60 minutes in the last seven days?’. Students doing at least 60 minutes of moderate to vigorous daily PA were considered physically active (Who, 2010). Time spent across TV was asked, and in data analysis, responses were grouped as >2 hours or ≤2 h (Canadian Society of Exercise Physiology, 2012). Time spent sleeping was evaluated with the ‘How many hours do you sleep on a normal school day?’ question, and answers were categorized as <9 h and ≥9 h (Hirshkowitz *et al.,* 2015).

The third layer (community networks) consisted of family and friends. Friends were examined in detail in the KID-KINDL questionnaire. Hence, we only included family structure (living with both parents/single parent) and the number of siblings (none, one sibling, and more than one sibling) as independent variables in this layer. The variable was dichotomized as having a sibling or not having a sibling for regression analyses.

The last layer (life conditions) included parents’ education, family income, neighborhood socioeconomic status (SES), and household characteristics. The parent with the highest education level was considered for the parents’ education. The groups were categorized as lower than high school/high school and above. For family income, children were to reveal their perception through three categories: expenses higher/equal/lower than our income. Then, the groups whose income was higher and equal to their expenses were combined for further analysis. The family income was also asked directly, but 61.1% of the children chose the ‘I do not know’ option for this variable. Thus, this variable was excluded. The neighborhood SES was also used as a measure. Children were to name the ‘mahalle’ (neighborhood) they lived in. The 24 neighborhoods of the Bayrakli district resemble different socioeconomic backgrounds, although this is not a well-documented classification. For this purpose, we used the land price (per meter square) lists of the Presidency of Revenue Management. We calculated the average land price for each neighborhood. The SES categories for each neighborhood were determined as three categories: good (higher than 1000 Turkish Lira-TL), moderate (602-1000 TL), and bad (lower than 602 TL).

We also asked about the characteristics of their household. Two questions were used to determine the number of persons per room: ‘Number of rooms except for the bathroom and kitchen’ and ‘Number of people living in your home’. The responses were divided into two categories: <1.5 and ≥1.5. The availability of a safe playground was asked, ‘Is there a space outside your house where you can play safely?’. With the question, ‘Do you have your own room?’ the presence of the child’s own room was discovered. The house’s structural features were also questioned for their inadequacy. ‘Is there a problem in the house you live in like; a leaking roof, damp roof/walls/foundation, rot in window frames or floor?’ question was asked for structural problems. Three questions about sanitation were asked: ‘Is there a bath/shower for the sole use of the household?’, ‘Is there an indoor flushing toilet for the sole use of the household?’, and ‘Is there hot running water in your household?’. If any of those questions were answered yes, we assumed there was a sanitation problem (Chzhen *et al.,* 2014).

### Data collection

Permission to collect data on the schools was taken from the Provincial Directorate of National Education, and ethical approval of this study was granted by the Ege University Clinical Research Ethics Committee (16-4/50). A pilot study was carried out before the research to evaluate the time needed and the reader friendliness of the form. It has been determined that one lesson hour was sufficient to answer the questions. Five questions and one explanation were changed for clarity. The study was explained to each of the 24 school principals, and the dates for data collection were arranged. Data were collected between April and June 2016 at the appointed time in a course hour by HD, and the informed consent of students was also approved. Data collection forms were filled out with self-reporting with anonymity and privacy. Students who had struggled filling out the forms were helped if they asked. Two students did not want to participate, one student had a mental disability, and 191 of them were not at school on the day of the research. A second tour for the missing data was not planned, as the data collection was already toward the end of the semester.

### Statistical analyses

The negatively oriented questions (questions 1, 2, 3, 6, 7, 8, 15, 16, 20, and 24) scores were reversed when computing the total score of the KID-KINDL questionnaire. The scores for each dimension were calculated and converted to a scale of 0 to 100. The total score was then calculated. In evaluating the relationship between QoL and independent variables, Student’s *t* test and Mann–Whitney *U* test, and Kruskal–Wallis *H* test were used. Templeton’s two-step normalization method for measurement-type variables was used for KINDL scores as it was not distributed normally (Templeton, 2011). Statistically significant variables were added to the multiple linear regression analysis in five different models. The first four models included variables in each layer of Dahlgren model to show their relationship with QoL alone. And a fifth model included all significant variables. *P* < 0.05 was accepted as the statistical significance level.

## Results

In the study, 1090 students participated. One hundred and ninety-one children who were not at school on the day of data collection were mainly enrolled in overcrowded public schools. All students from private schools took part in data collecting. The mean age was 12.5 ± 0.5. The mean QoL score was 71.3 ± 12.6. The highest QoL scores were obtained from the friend dimension, and the lowest was from the school dimension (Table 1). All variables except neighborhood SES had a statistically significant relationship with KINDL scores. The characteristics of the sample and their relation to KINDL scores of children are summarized in Table 2.

**Table 1.** Quality of life scores of children (*n* = 1078)

	Mean ± SS	Min–Max
Physical well-being	69.5 ± 18.7	6.3–100.0
Emotional well-being	73.0 ± 17.8	6.3–100.0
Self-esteem	63.5 ± 23.3	0–100.0
Family	81.4 ± 16.5	6.3–100.0
Friends	81.9 ± 17.2	0–100.0
School	58.6 ± 19.6	0–100.0
Total QoL score	71.3 ± 12.6	25.0–97.9

**Table 2.** Characteristics of the sample and their relation with KINDL scores of children

	Variable	N	%	KINDL score	*P*
Basic features	Sex				
	Female Male	514 548	48.4 51.6	70.0 ± 13.6 72.6 ± 11.5	***P*** **= 0.001**
	Health status				
	Very well Good Bad	692 352 30	64.4 32.8 2.8	75.3 ± 10.5 64.8 ± 12.4 54.5 ± 14.5	***P*** **< 0.001** ^1^
	Academic achievement				
	Close to the top In the middle Close to the bottom	534 432 120	49.2 38.8 11.0	74.5 ± 11.8 69.4 ± 12.4 64.1 ± 12.7	***P*** **< 0.001** ^1^
Indıvidual lifestyle factors	Body perception				
	Underweight Normal Overweight	231 551 295	21.4 51.2 27.4	71.5 ± 12.4 72.9 ± 12.3 68.4 ± 12.7^2^	***P*** **< 0.001**
	Physical activity				
	Active Inactive	228 822	21.7 78.3	74.1 ± 11.6 70.6 ± 12.7	***P*** **< 0.001**
	Time spent watching the TV				
	≤2 h >2 h	724 348	67.5 32.5	72.0 ± 13.0 69.9 ± 11.8	***P*** **= 0.010**
	Time spent sleeping				
	<9 h ≥9 h	545 468	53.8 46.2	69.9 ± 12.8 72.9 ± 12.4	***P*** **< 0.001**
Community networks	Family structure				
	Living with both parents Living with only one parent	965 103	90.4 9.6	71.7 ± 12.6 68.6 ± 11.7	***P*** **= 0.021**
	Number of siblings				
	No sibling One sibling More than one sibling	126 502 448	11.7 46.7 41.6	72.7 ± 12.5 72.1 ± 12.1^3^ 70.0 ± 13.1^3^	***P*** **= 0.040**
Living conditions	Highest parental education				
	<High school ≥High school	500 538	48.2 51.8	70.2 ± 12.6 72.2 ± 12.6	***P*** **= 0.011**
	Family income				
	Income > expenses Income = expenses Income < expenses	244 632 191	22.9 59.2 17.9	72.1 ± 13.1 72.1 ± 12.1 66.9 ± 12.9^2^	***P*** **< 0.001**
	Neighborhood SES				
	High Middle Low	221 166 573	23.0 17.3 59.7	72.1 ± 12.7 72.4 ± 12.9 70.4 ± 12.8	*P* = 0.052
	Number of people per room				
	<1.5 ≥1.5	738 316	70.0 30.0	72.2 ± 12.2 69.4 ± 13.4	***P*** **= 0.001**
	Safe playground				
	Available, yes Not available, no	800 258	75.6 24.4	72.8 ± 12.1 66.7 ± 13.3	***P*** **< 0.001**
	Child’s own room				
	Present No	761 313	70.9 29.1	72.9 ± 12.0 67.4 ± 13.1	***P*** **< 0.001**
	Structural problems in the household				
	Exist Do not exist	956 113	89.4 10.6	72.4 ± 12.1 62.3 ± 13.4	***P*** **< 0.001**
	Sanitation problems in the household				
	Exist Do not exist	1007 62	88.2 11.8	71.9 ± 12.1 61.5 ± 16.8	***P*** **< 0.001**

*Note*. *Post hoc* analysis results:

1 All groups are significantly different from each other.

2 Groups is significantly different than others.

3 Groups are significantly different from each other.

Results showed that QoL scores increased as perceived health status and academic achievement improved. There was no difference between underweight and normal body perception groups for QoL, but overweight children had significantly lower scores than the other groups. The physically active children who spent less time across TV and more time sleeping had higher QoL scores.

Children living with both of their parents had higher QoL scores. We found no difference between having a sibling or being an only child. However, among the children who had siblings, those who just had one sibling had higher QoL scores.

For the living conditions layer, parents’ higher education levels are related to higher QoL scores. Children who thought their income was less than their expenses and those who lived in more crowded homes had lower QoL scores. While having a safe playground and a child’s room at home improved QoL significantly, any structural or sanitation issues were associated with lower scores.

Multiple linear regression was conducted to show each layer’s relationship with QoL (*P* < 0.001). According to the *R* ^2^ values of each level associated with regression models, basic features (Model 1), individual lifestyle factors (Model 2), community networks (Model 3), and living conditions (Model 4) account for 24.4%, 5.2%, 1.2%, and 5.8%, respectively, of the variation in our study. Model 5, which included all statistically relevant variables, explained 31.7% of the variance in QoL scores. And all significant variables remained significant except PA, the number of siblings, parental education status, the number of persons per room, and household sanitation concerns. These two variables stand out in the analysis results. First, QoL scores dropped 7.8 points as health perceptions got worse. Additionally, any structural issue in the home reduced QoL by 6.6 points (Table 3).

**Table 3.** Association between KINDL scores and layers of determinants of health in five models

		Model 1		Model 2		Model 3		Model 4		Model 5	
		B	***P***	B	***P***	B	***P***	B	***P***	B	***P***
Basic features	Sex (ref:male)	−2.196	**0.001**							−1.399	**0.056**
	Health status^1^	−9.240	**<0.001**							−7.766	**<0.001**
	Academic achievement^2^	−4.238	**<0.001**							−3.914	**<0.001**
Indıvidual lifestyle factors	Body perception (ref: normal)			−3.370	**<0.001**					−2.167	**0.007**
	Physical activity (ref: active)			3.385	**<0.001**					1.493	0.094
	Time spent watching TV (ref:<2 h)			−2.265	**0.007**					−1.537	**0.044**
	Time spent sleeping (ref:<9 h)			2.826	**<0.001**					2.107	**0.003**
Community networks	Family structure (ref: both parents)					−3.215	**0.014**			−2.509	**0.041**
	Number of siblings (ref: ≤1 sibling)					−2.095	**0.007**			1.015	0.211
Living conditions	Highest parental education (ref:<high school)							0.454	0.626	−0.566	0.531
	Family income (ref:income≥expenses)							−3.118	**0.002**	−2.926	**0.002**
	Number of people per room (ref:<1.5)							0.590	0.509	1.107	0.225
	Safe playground (ref:Has)							−3.735	**<0.001**	−2.277	**0.009**
	Child’s own room (ref:Has)							−3.792	**<0.001**	−1.772	**0.038**
	Structural problems in the household (ref: Has)							−8.116	**<0.001**	−6.605	**<0.001**
	Sanitation problems in the household (ref: Has)							−5.025	**0.003**	−2.429	0.127
	*R* ^2^ and *P* values	***R*** ^2^ **= 0.244** ***P*** **< 0.001**		***R*** ^2^ **= 0.052** ***P*** **< 0.001**		***R*** ^2^ **= 0.012** ***P*** **= 0.002**		***R*** ^2^ **= 0.132** ***P*** **< 0.001**		***R*** ^2^ **= 0.317** ***P*** **< 0.001**	

1 Variable codded as 1 – very well, 2 – good, and 3 – bad.

2 Variable codded as 1 – close to the top, 2 – in the middle, and 3 – close to the bottom.

## Discussion

In this study, we considered the relation of different layers of Dahlgren’s scheme of health determinants with QoL in children. We found that being a boy, having better health, better academic achievement, and normal/thin body perception were related to higher scores of QoL. Children who were physically more active and had better sleeping habits had higher scores. Living with both parents increased QoL, while having more than one sibling had a worsening effect. The physical problems found in the home had a detrimental impact on the QoL as well. The four layers’ relationships with children’s QoL were most strongly influenced by the basic characteristics (sex, health status, and academic achievement), which accounted for the majority of the variation in our model.

### Basic characteristics

Our findings were consistent with earlier research on girls’ lower QoL scores (Michel *et al.,* 2009; Rezende *et al.,* 2017). This could be due to pubertal changes, which occur earlier in girls than in boys in this age group. The questionnaire explains health-related QoL in the first place, and perception of health has a profound association with QoL (Sawatzky, 2007; Rezende *et al.,* 2017). School achievement was revealed to be an important determinant of QoL, and better school achievement was associated with higher QoL levels, similar to prior studies (Altıparmak, 2010; Ak, 2014; Rezende *et al.,* 2017). The education system produces stress through exam anxiety, and most parents pressure their children to perform in school.

### Lifestyle factors

Children who perceived themselves as overweight had lower health-related QoL scores. A recent study from China showed children who were satisfied with their bodies had significantly higher scores (Liu *et al.,* 2019). Two other studies from Norway and Italy reported body perception of being overweight is related to lower scores (Haraldstad *et al.,* 2011; Petracci and Cavrini, 2013). In recent years, the perception of beauty in the media has caused children to perceive their bodies as overweight. It is thought that the negative emotions created by this situation are related to lower QoL scores.

Sedentary behaviors like physical inactivity and spending more time on TV, as well as poor sleeping time, were found to decrease QoL significantly in the binary analysis. In the regression analysis, PA activity was not found to be associated. We lost significance after regression, possibly because other variables referring to sedentary behavior had stronger correlations with QoL. Hoare *et al*. found a similar relationship between PA and QoL in Australia as in Malaysia and Istanbul; children with more PA had better QoL (Ak, 2014; Wafa *et al.,* 2016; Hoare *et al.,* 2019). In a recent study in Spain, sedentary behaviors and PA were found to have a significant relationship with QoL; however, while moderate to vigorous PA was related with higher QoL scores in males, light PA was associated with higher QoL scores in females (Ávila-García *et al.*, 2021). Another study questioned BMI, sleeping habits, physical inactivity, and screen hours and reported that these variables associated with BMI had a strong relationship with QoL (Chen *et al.,* 2014). In children, sedentary behaviors and lack of PA can cause obesity and health problems and reduce children’s communication with their social environment. Children’s exposure to inappropriate content on television or the internet can negatively impact their psychology. Spending long hours watching TV can reduce school achievement. And all these things can cause lower QoL in children.

### Community networks

Although community networks were not thoroughly evaluated in this study, the findings revealed that living in separated families has a negative effect on children’s QoL (Ak, 2014; Houben-van Herten *et al.,* 2015). The absence of one of the parental figures in the house negatively affects the child’s psychology and reduces the QoL. Botelho Guedes *et al.* reported in Portuguese that living with both parents and having a better family connection is associated with higher QoL scores (Botelho Guedes *et al.*, 2022). Those with one brother or sister appear to have a better QoL than those with multiple siblings. An increase in the number of children in the family may reduce the family’s economic and emotional share (Wu *et al.,* 2015; McCracken *et al.,* 2016). When this variable is adjusted with other determinants, the relation disappears, thus revealing that this variable may interact with other socioeconomic determinants in the household.

### Life conditions

In contrast to binary analysis, we found no significant association between parental education and QoL in regression. There are different results in the literature; a study in seven European countries found a significant relationship, while studies in the Netherlands and Istanbul found the opposite (von Rueden *et al.,* 2006; Ak 2014; Houben-van Herten *et al.,* 2015). Children who thought their family’s income was less than their costs scored significantly lower. It is well known that poverty affects the QoL due to its effects on a child’s physical and psychosocial environment (Cohen *et al.,* 2010). SES status was regarded as low in Dutch children if income was less than 2000 Euros and was found to be associated with lower QoL ratings (Hassan *et al.*, 2021). Our study found that 61.1% of children cannot express family income as a numerical determinant but can reveal a subjective perception. As a result, income perception is a better tool for children to understand the household’s income level. This is a valuable interpretation for studies attempting to gather such data from children.

We found no significant relationship between neighborhood SES and QoL, which contradicted prior research that suggested that neighborhood SES has a crucial influence on children’s mental health, learning, academic achievement, and social and emotional outcomes (Drukker *et al.,* 2003; Villanueva *et al.,* 2016). In the study, the categorization of the neighborhood SES based on average land price may have been insufficient. Also, the neighborhoods’ heterogeneity may have distracted this categorization. The insignificant relationship may have resulted from biased categorization rather than an absence of such a relationship.

Among household characteristics, those with statistical significance in bivariate analysis (having a safe playground and a child room in the house) remained significant in regression analyses after adjustment for other socioeconomic variables. Only a few studies examine the relationship between children’s household conditions and QoL. A study in Izmir reported QoL affected by the presence of a child room (Altıparmak, 2010). In Edinburgh, researchers found as green land use increases, QoL increases (Schreier and Chen, 2013). Structural and sanitation problems show extreme poverty, and structural problems were significant in our analyses. All these results may indicate that not only the poverty but also the negative consequences directly related to children affects the QoL of a child.

### Strengths and weaknesses of the study

Our study results from the Bayrakli district could give an idea about the children living in developed and urban districts of other middle-income countries. This is significant because research about urban children’s health in such a broad and structured framework is rare in such countries. Their significance to QoL needs to be adequately investigated. We were able to assess the components of children’s daily lives comprehensively thanks to the application of Dahlgren’s model, which provided a strong rationale for determinants of health. Many aspects of a child’s life were taken into consideration. Various variables have been used to overcome obstacles in measuring children’s socioeconomic environments.

Some limitations have to be taken into consideration as well. First, the cross-sectional study design precludes causal inference. Even though there was a good representation of the Bayrakli district, the study population resembles the developed and modern parts of the country, which does not imply country-specific results. The self-reported nature of the questionnaire and questions based on perception hold the possibility of reporting bias. Anonymity was guaranteed, but the data collection administered in the classrooms may have caused some children to hesitate to fill out the truth because of their peers’ attendance nearby.

In conclusion, health, success, physical appearance, and wealth were found to be significantly associated with QoL in this study, demonstrating the importance of children’s perceptions on their lives. Individual lifestyle variables have been shown to influence QoL as early as childhood. Adopting interventions during this period may also benefit adult health. It was also significant to discover that the socioeconomic environment in which the child interacts are strongly linked to the QoL. These characteristics may serve as a roadmap for future initiatives to increase children’s QoL.
